# How defective mitochondrial electrical activity leads to inherited blindness

**DOI:** 10.1073/pnas.2315649120

**Published:** 2023-10-25

**Authors:** Peter J. Burke

**Affiliations:** ^a^Department of Electrical Engineering and Computer Science, University of California, Irvine, CA 92697; ^b^Department of Biomedical Engineering, University of California, Irvine, CA 92697; ^c^Department of Chemical and Biomolecular Engineering, University of California, Irvine, CA 92697; ^d^Department of Materials Science and Engineering, University of California, Irvine, CA 92697

How does a genetic defect in an enzyme complex that generates an electrical voltage across the inner membrane of mitochondria cause the inherited blindness disease Leber’s Hereditary Optic Neuropathy (LHON)? This mystery has plagued medicine for over three decades ever since Wallace et al. (1) discovered, through careful genetic analysis of patient family trees and Sanger sequencing of patient mitochondrial DNA (mtDNA), that a specific mtDNA mutation is the cause of LHON, hence founding the now burgeoning field of mitochondrial medicine (2). Since then, researchers have identified multiple similar mtDNA mutations that cause LHON (3). These defects share a common feature: They are in regions that encode for the membrane proteins that create the voltage across the mitochondrial inner membrane, using the chemical energy stored in sugars and fats to drive the process. In PNAS (4), Fuller et al. take an important step to answer the question of how this genetic mtDNA defect leads to LHON and causes blindness. Although this deals with only one disease, LHON has become the canonical, marquee example of how mitochondrial function impacts multiple (possibly all) diseases, since it involves all organs. Thus, the work by Fuller et al. in PNAS (4), and similar work addressing LHON, while addressing only one disease, has broad significance as a model for dissecting the link between energy, health, and life.

**Fig. 1.**
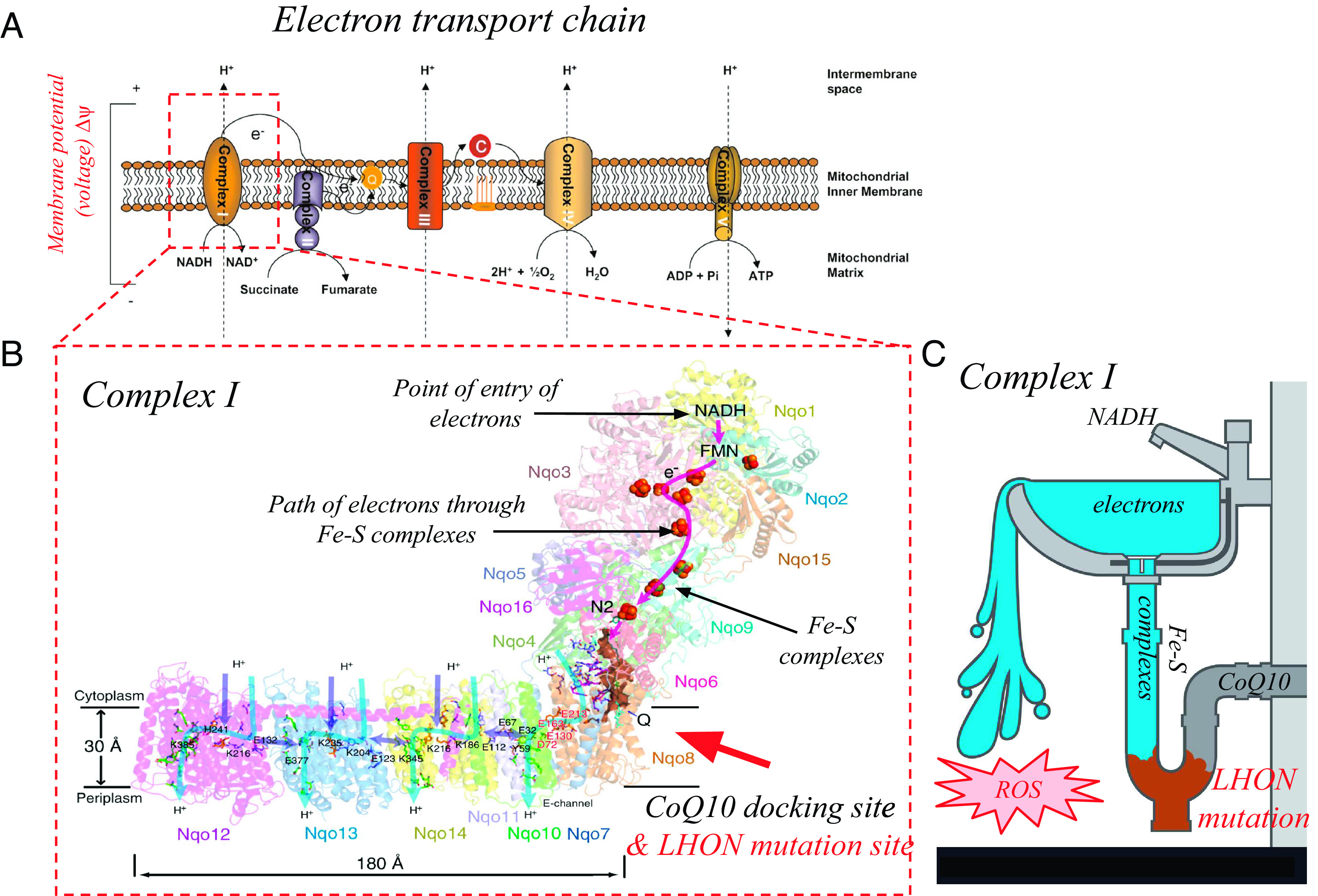
(*A*) Schematic and functional diagram of the electron transport chain complexes I–V on the mitochondrial inner membrane. (*B*) Structural and functional detail of complex I, showing the LHON mutation site and its relationship to the path electrons traverse from the initial oxidation of NADH by CI to the final reduction of CoQ10 by CI. (*C*) Cartoon analog of the finding of Fuller et al. (4). *A* and *B* adopted from refs. 5 and 6, with permission.

In order to more carefully answer this question, it is necessary to dive deeper into the detailed electrical machinery inside the mitochondria organelle (Fig. 1). Four protein complexes (CI–IV) in the mitochondrial inner membrane pump protons across the membrane to create a voltage and pH gradient. This voltage is used by a fifth complex (CV, F_o_F_1_-ATP synthase) to drive ATP synthesis from ADP. Thus, the mitochondria act as a battery by converting chemical energy stored in sugars and fats to stored electrical energy. The stored electrical energy is converted back to chemical energy in the form of ATP by CV. It is in the details of how the four complexes create the voltage that problems can arise.

The work by Fuller et al. in PNAS, and similar work addressing LHON, while addressing only one disease, has broad significance as a model for dissecting the link between energy, health, and life.

In the initial steps of the process (Fig. 1*B*), CI and CII oxidize substrates NADH and succinate, which are generated upstream in the Krebs cycle. The transferred electrons make their way through CI and CII (stepping down an energy “ladder”) to a terminal location on each complex, at which point they reduce the lipid-soluble quinone Coenzyme Q10 (CoQ10). After reduction, CoQ10 diffuses through the membrane to CIII, where the electron is transferred to CIII. CIII then reduces the soluble protein cytochrome C (CytC), which then travels to reduce CIV, which in turn reduces molecular oxygen to water, i.e., respires. The redox reactions at CI–CIV are energetically and chemically coupled to the process of pumping of protons across the membrane, which creates the mitochondrial voltage Δψ and pH gradient, together referred to as the electrochemical potential Δμ = Δψ − [2.3(RT/F)] ΔpH, with R the universal gas constant, T the absolute temperature, and F the Faraday constant. As the LHON defect occurs in Complex I, it is of special interest.

Complex I is a large complex with multiple subunits (5, 7). The subunits are coded by both nuclear DNA (39 subunits), and more importantly in this context mtDNA (7 subunits). Although Wallace in 1988 identified a single genetic defect encoding subunit 4, since then multiple additional defects have been identified (3), and more are likely to be found in the future. Fuller et al. in PNAS (4) focused in their work on the most deleterious of the defects m.3460 G > A in ND1, resulting in the replacement of alanine A52 with a threonine, since it is most common and gives rise to the most severe clinical pathologies.

As shown in Fig. 1*B*, NADH reduces a flavin near the tip of the CI arm. The electrons then physically move through the arm, gradually losing energy, via hopping through several Fe–S complexes, until they reach the final Fe–S complex. At this site, CoQ10 diffuses in from the bilayer, docks, is reduced, and then is released to continue its journey to CIII. Careful biochemical analysis of CI efficiency through respiration studies on cells, isolated mitochondria, cybrids (hybrids of host cells and LHON patient mitochondria) (8, 9), and mouse models (10) all have shown a moderate reduction in the capacity of CI to pump protons (and therefore degraded ability to create a membrane voltage), which ultimately leads to lower ATP synthesis ability of the cell, a possible contributing factor to blindness in LHON.

A more significant impact of the LHON defect is the generation of additional reactive oxygen species (ROS) by the mitochondria. Researchers have shown that ROS is increased in cells from patients as well as cybrids carrying the LHON mutation (11). It is generally believed that ROS is one signaling mechanism that can trigger apoptosis. In the genetically inherited disease LHON, patients (typically males) experience sudden onset of blindness in both eyes in young adulthood at around age 20 (3). The ~ million retinal ganglion cells (RGC) that all die during LHON onset are believed to die via apoptosis (3). This paints a picture of excess ROS generated by defects in CI as the cause of the apoptotic cascade in the RGC cells, resulting in blindness. However, until the work of Fuller et al. in PNAS (4), the mechanism by which the CI defect increases ROS was not known.

Two clues led Fuller et al. in PNAS (4) to focus on the site where CoQ10 is reduced by CI. First, the rate-limiting factor in the CI process is known to be the final step, that is the reduction of CoQ10 by CI. Second, the physical location of the 3460 defect is an amino acid close to this site on CI. Using the best of modern tools in molecular dynamics (MD) simulations, Fuller et al. find that the defect causes a reduced ability of CoQ10 to exit the docking portion of CI after it is reduced. Their explanation is that the larger amino acid threonine protrudes into the docking pocket where CoQ10 must escape from once reduced. In fact, Fuller et al. found the ability of reduced CoQ10 to be released by CI to be one billion times slower in the mutated CI as compared to the wild-type CI. This would cause the electrons upstream in the arm of the CI to leak out since they have nowhere to go. Fuller et al. hypothesize that this causes significant additional ROS generation, as the out-of-place electrons reduce molecular oxygen, and other possible targets, instead of being passed onto the CoQ10, creating excess ROS as observed in other experiments (8–10). Thus, Fuller et al. have proposed the mechanism (backed by MD simulations) by which LHON defects cause increased ROS.

An analogy is when the garbage disposal in a kitchen sink becomes clogged and the water from the faucet is left running (Fig. 1*C*). One doesn’t know exactly what is going to happen, but it surely will be trouble for the whole house, short term and long term. In the case of LHON, the short-term trouble is increased ROS production due to electron leakage that otherwise would have been guided through the complex to reduce CoQ10. The long-term trouble is the adult onset blindness. Fuller et al. in PNAS (4) have figured out, at the molecular level, how and why the sink gets clogged.

Many questions still remain unanswered (12). How exactly does increased ROS cause apoptosis? Why does it occur in early adulthood? Why does it occur only in the eye in LHON patients? It is likely the ROS alone does not cause the apoptotic wave resulting in ~ one million cells dying, but that it makes neurons in the eye more likely to undergo apoptosis when confronted with additional, unknown apoptotic signals, perhaps related to age or environmental factors. Experiments with LHON cybrids have confirmed they are more prone to apoptosis (13, 14). One important clue is that the axons in the eye are unmyelinated, a requirement for optical transparency in the organ. This causes increased energetic needs to restore the action potential in comparison to myelinated neurons (3) and may leave them more vulnerable to apoptosis. Furthermore, many of the regions of the axon are mitochondria rich (3), leading to possibly increased ROS in these regions. In sum, the intense energetic needs to drive the action potentials during vision lead to anatomical features in the eye which may be more vulnerable to ROS-influenced or induced apoptosis in times of additional stress or other signals.

This is just the tip of the iceberg in our understanding of structure–function relationships in the mitochondria electrophysiology and its impact on pathology and health. While “omics”-type approaches have an important role to play (15), this important example illustrates how such a “list of parts” approach will not be sufficient to determine the functional impact of mitochondria on health and disease. Functional screening (as opposed to content or structure), specifically of mitochondrial electrical activity (16–18), is critical to understand the impact of genetic and structural features energy producing organelles on human health. There is even a potential role of quantum properties, as quantum spin plays a critical role in ROS chemistry, with evidence for roles of quantum spin the in biology of the eye (19) and mitochondria (20). Combining both approaches (structure and function) will enable progress in the growing field of the connection between energy and life in human health and could provide avenues to explore for therapies not just for diseases of the eye but for all energy-related diseases, including aging itself (21).
